# Clinical, biochemical and genetic spectrum of 70 patients with ACAD9 deficiency: is riboflavin supplementation effective?

**DOI:** 10.1186/s13023-018-0784-8

**Published:** 2018-07-19

**Authors:** Birgit M. Repp, Elisa Mastantuono, Charlotte L. Alston, Manuel Schiff, Tobias B. Haack, Agnes Rötig, Anna Ardissone, Anne Lombès, Claudia B. Catarino, Daria Diodato, Gudrun Schottmann, Joanna Poulton, Alberto Burlina, An Jonckheere, Arnold Munnich, Boris Rolinski, Daniele Ghezzi, Dariusz Rokicki, Diana Wellesley, Diego Martinelli, Ding Wenhong, Eleonora Lamantea, Elsebet Ostergaard, Ewa Pronicka, Germaine Pierre, Hubert J. M. Smeets, Ilka Wittig, Ingrid Scurr, Irenaeus F. M. de Coo, Isabella Moroni, Joél Smet, Johannes A. Mayr, Lifang Dai, Linda de Meirleir, Markus Schuelke, Massimo Zeviani, Raphael J. Morscher, Robert McFarland, Sara Seneca, Thomas Klopstock, Thomas Meitinger, Thomas Wieland, Tim M. Strom, Ulrike Herberg, Uwe Ahting, Wolfgang Sperl, Marie-Cecile Nassogne, Han Ling, Fang Fang, Peter Freisinger, Rudy Van Coster, Valentina Strecker, Robert W. Taylor, Johannes Häberle, Jerry Vockley, Holger Prokisch, Saskia Wortmann

**Affiliations:** 1 Institute of Human Genetics, Technische Universität München, Trogerstrasse 32, 81675 Munich, Germany; 2 Institute of Human Genetics, Helmholtz Zentrum München, Munich, Germany; 3 Wellcome Centre for Mitochondrial Research, Newcastle University, Newcastle upon Tyne, UK; 4 UMR1141, PROTECT, INSERM, Université Paris Diderot, Sorbonne Paris Cité, 75019 Paris, France; 5 Reference Center for Inborn Errors of Metabolism, Robert Debré University Hospital, APHP, 75019 Paris, France; 6 UMR1163, Université Paris Descartes, Sorbonne Paris Cité, Institut IMAGINE, 24 Boulevard du Montparnasse, 75015 Paris, France; 7 Unit of Molecular Neurogenetics, Fondazione Istituto Neurologico “Carlo Besta”, Milan, Italy; 8 Child Neurology, Fondazione IRCCS Istituto Neurologico “Carlo Besta”, Milan, Italy; 9 Department of Molecular and Translational Medicine DIMET, University of Milan-Bicocca, Milan, Italy; 10 INSERM U1016, Institut Cochin, Paris, France; 11 Department of Neurology, Friedrich-Baur-Institute, University Hospital of the Ludwig-Maximilians-Universität München, Munich, Germany; 12 Muscular and Neurodegenerative Disorders Unit, Bambino Gesu´ Children’s Hospital, IRCCS, Rome, Italy; 13 NeuroCure Clinical Research Center (NCRC), Charité-Universitätsmedizin Berlin, Corporate member of Freie Universität Berlin, Humboldt-Universität zu Berlin, and Berlin Institute of Health (BIH), Berlin, Germany; 14 Nuffield Department of Women’s and Reproductive Health, University of Oxford, The Women’s Centre, John Radcliffe Hospital, Oxford, UK; 15 Division of Inherited Metabolic Diseases, Department of Paediatrics, University Hospital of Padova, Padova, Italy; 16 Department of Pediatrics, Antwerp University Hospital, Edegem, Belgium; 17 ELBLAB GmbH, Riesa, Germany; 18 Department of Pathophysiology and Transplantation, University of Milan, Milan, Italy; 19 Department of Pediatrics, Nutrition and Metabolic Diseases, The Children’s Memorial Health Institute, Warsaw, Poland; 20 Wessex Clinical Genetics Service, Princess Anne Hospital, Southampton, UK; 21 Genetics and Rare Diseases Research Division, Unit of Metabolism, Bambino Gesù Children’s Research Hospital, Rome, Italy; 22 Department of Pediatric cardiology, Beijing Anzhe Hospital, Captital Medical University, Beijing, China; 23 Department of Clinical Genetics, Copenhagen University Hospital Rigshospitalet, Blegdamsvej 9, 2100 Copenhagen, Denmark; 24 South West Regional Metabolic Department, Bristol Royal Hospital for Children, Bristol, BS1 3NU UK; 25 Department of Genetics and Cell Biology, Maastricht University Medical Centre, Maastricht, The Netherlands; 26 Functional Proteomics, SFB 815 Core Unit, Faculty of Medicine, Goethe-University, Frankfurt am Main, Germany; 27 Department of Clinical Genetics, St Michael’s Hospital, Bristol, UK; 28 Department of Neurology, Erasmus MC, Rotterdam, Netherlands; 29 Department of Clinical Genetics, Research School GROW, Maastricht University Medical Centre, Maastricht, The Netherlands; 30 Department of Pediatric Neurology and Metabolism, Ghent University Hospital, De Pintelaan, Ghent, Belgium; 31 Department of Pediatrics, Salzburger Landeskliniken (SALK) and Paracelsus Medical University (PMU), Salzburg, Austria; 32 Department of Neurology, Beijing Children’s Hospital, Capital Medical University, National Center for Children’s Health, Beijing, China; 33 Research Group Reproduction and Genetics, Vrije Universiteit Brussel, Brussels, Belgium; 34 Department of Pediatric Neurology, UZ Brussel, Vrije Universiteit Brussel, Brussels, Belgium; 35 MRC-Mitochondrial Biology Unit, Cambridge, Cambridgeshire UK; 36 Division of Human Genetics, Medical University Innsbruck, Innsbruck, Austria; 37 Center for Medical Genetics, UZ Brussel, Research Group Reproduction and Genetics (REGE), Vrije Universiteit Brussel, Brussels, Belgium; 38 German Center for Neurodegenerative Diseases (DZNE), Munich, Germany; 39 Munich Cluster of Systems Neurology (SyNergy), Munich, Germany; 40 DZHK (German Centre for Cardiovascular Research), partner site Munich Heart Alliance, Munich, Germany; 41 Department of Pediatric Cardiology, University of Bonn, Bonn, Germany; 42 Université Catholique de Louvain, Cliniques Universitaires Saint-Luc, Brussels, Belgium; 43 Department of Pediatrics, Klinikum Reutlingen, Reutlingen, Germany; 44 Division of Metabolism and Children’s Research Center, University Children’s Hospital, Zurich, Switzerland; 45 Department of Pediatrics, University of Pittsburgh School of Medicine, University of Pittsburgh, Children’s Hospital of Pittsburgh of UPMC, Pittsburgh, USA; 46 Institute of Medical Genetics and Applied Genomics, University of Tübingen, Tübingen, Germany

**Keywords:** Complex I, Cardiomyopathy, Heart transplantation, Mitochondrial disorder, Lactic acidosis, Treatment, Prognosis, Neonatal, Vitamin, Activities of daily living

## Abstract

**Background:**

Mitochondrial acyl-CoA dehydrogenase family member 9 (ACAD9) is essential for the assembly of mitochondrial respiratory chain complex I. Disease causing biallelic variants in *ACAD9* have been reported in individuals presenting with lactic acidosis and cardiomyopathy.

**Results:**

We describe the genetic, clinical and biochemical findings in a cohort of 70 patients, of whom 29 previously unpublished. We found 34 known and 18 previously unreported variants in *ACAD9.* No patients harbored biallelic loss of function mutations, indicating that this combination is unlikely to be compatible with life. Causal pathogenic variants were distributed throughout the entire gene, and there was no obvious genotype-phenotype correlation.

Most of the patients presented in the first year of life. For this subgroup the survival was poor (50% not surviving the first 2 years) comparing to patients with a later presentation (more than 90% surviving 10 years). The most common clinical findings were cardiomyopathy (85%), muscular weakness (75%) and exercise intolerance (72%). Interestingly, severe intellectual deficits were only reported in one patient and severe developmental delays in four patients. More than 70% of the patients were able to perform the same activities of daily living when compared to peers.

**Conclusions:**

Our data show that riboflavin treatment improves complex I activity in the majority of patient-derived fibroblasts tested. This effect was also reported for most of the treated patients and is mirrored in the survival data. In the patient group with disease-onset below 1 year of age, we observed a statistically-significant better survival for patients treated with riboflavin.

**Electronic supplementary material:**

The online version of this article (10.1186/s13023-018-0784-8) contains supplementary material, which is available to authorized users.

## Background

Complex I of the mammalian mitochondrial respiratory chain is a large multimeric complex composed of 44 subunits encoded by the mitochondrial and nuclear genome. Beside the structural subunits, at least 19 complex I specific assembly factors are required to obtain fully assembled complex I [1].

One assembly factor is ACAD9. Beside its role in the proper assembly of complex I, ACAD9 exhibits acyl-CoA dehydrogenase (ACAD) activity [2, 3]. ACADs belong to a family of flavoenzymes involved in the ß-oxidation of acyl-CoA and amino acid catabolism. ACAD9 is most homologous (47% amino acid identity, 65% amino acid similarity) to very long-chain acyl-CoA dehydrogenase (VLCAD). Both ACAD9 and VLCAD function as homodimers associated with the inner mitochondrial membrane and catalyze the initial step of the fatty acid oxidation (FAO) cycle [4].

Mutations in *ACAD9* have been related to human disease [5–7]. The clinical presentation of ACAD9 deficiency is dominated by cardiomyopathy. Other features are lactic acidosis, myopathy and developmental delay. Age of onset, severity of symptoms and progression are variable. We have shown that residual ACAD9 enzyme activity, and not complex I activity, correlates with the severity of clinical symptoms in ACAD9 deficient patients [3].

In anecdotal reports of patients with a predominance of myopathic features, alleviation of symptoms under riboflavin treatment has been reported [5, 7, 8]. Riboflavin is the precursor of flavin adenine dinucleotide (FAD) and flavin mononucleotide (FMN), which are cofactors for complex I and numerous dehydrogenases involved in FAO. The mode of action is unclear, previous studies suggested that riboflavin increases the mitochondrial FAD concentration thereby supporting FAD binding and consecutively improving ACAD9 folding and stability, thus promoting complex I assembly [9].

Bezafibrate, a peroxisome proliferator-activated receptor (PPAR)-alpha activator that controls the expression of many FAO genes, has been reported as a potential treatment for FAO disorders, with beneficial response in six patients [10]. Recently, this was weakened by a double-blind randomized crossover study of bezafibrate in five individuals with acyl-CoA dehydrogenase very long chain (ACADVL) deficiencies in whom no improvement could be detected [11].

In this study, we provide a comprehensive overview of the clinical, biochemical and genetic spectrum of 70 ACAD9 deficient individuals, of whom 29 are unpublished. We further evaluate the effect of riboflavin in patients and the effect of riboflavin and bezafibrate supplementation in patient-derived fibroblast cell lines.

## Methods

### Individuals

All procedures followed were in accordance with the ethical standards of the responsible committee on human experimentation (institutional and national) and with the Helsinki Declaration of 1975, as revised in 2000. Written informed consent was obtained from all individuals or caregivers. The clinical data were collected via an online survey completed by the respective physician. The online survey included 93 questions regarding age at presentation, current age or age at death, signs and symptoms during the fetal and neonatal period, at the beginning and during the course of disease, circumstances of death etc.. A special emphasis lay on the cardiac and neurological phenotype, daily life activities and the use of cardiac medication as well as vitmains and co-factors (e.g. riboflavin).

Kaplan Meier curves were created using the R project for statistical computing (survival package, https://www.r-project.org/).

### Molecular genetic investigations

Exome sequencing, panel sequencing and Sanger sequencing was performed as described previously [7, 12–22].

### Cell culture

Human fibroblast cells were grown in Dulbecco’s modified Eagle medium-high glucose supplemented with 10% fetal bovine serum, 1% penicillin-streptomycin (Invitrogen) and 200 μM uridine (Sigma-Aldrich) at 37 °C in an atmosphere containing 5% CO_2_.

### Riboflavin and bezafibrate treatment

The fibroblast cell lines were treated with 400 μM bezafibrate, 530 nM riboflavin or vehicle (DMSO) for 72 h as previously described [23, 24]. On the second day the cells were seeded at 20,000 cells/well in 80 μl DMEM in 96 well cell culture microplate and incubated overnight at 37 °C and 5% CO_2_. On the third day of the experiment the medium was changed to 180 μl unbuffered DMEM and incubated for at least 30 min at 37 °C without CO_2_.

### Oxygen consumption measurement

Oxygen consumption rate (OCR) was measured using an XF96 extracellular flux analyzer (Seahorse Biosciences, North Billeric, MA, USA) as previously described [25, 26] under basal conditions, in the presence of oligomycin (1 μM, ATP synthase inhibitor), FCCP (0.4 μM, mitochondrial oxidative phosphorlyation system (OXPHOS) uncoupler) antimycin A (2.5 μM, complex III inhibitor) and/or rotenone (0.5 μM, complex-I inhibitor). Antimycin and/or rotenone blocked all mitochondrial respiration and were subtracted from all values. Data was normalized to DNA content with CyQuant (Invitrogen).

### Western blot and BN-PAGE analysis

Western Blot analyses of different proteins were performed according to standard protocols [5, 27] ACAD9, VLCAD, MCAD, subunits of the respiratory chain complex I (NDUFS1, NDUFA9) and complex II (SDHA) were investigated and ß-actin was used as a loading control (Abcam, Sigma-Aldrich, MitoSciences 1:1000).

*Electrophoresis and in-gel quantification of fluorescent-labeled proteins* as well as *supercomplex assembly* are described in Additional file 1 [28, 29].

## Results

### Individuals

Seventy individuals (41 females) from 50 families were recruited, of which 29 were previously unreported (Additional file 2: Table S1 [30–32] and Additional file 3: Table S2). Individuals were numbered I1-I70, their respective fibroblast cell lines (if available) accordingly F1-F70. In the majority of patients investigated (*n* = 55) a complex I deficiency was found in sceletal (*n* = 44) or heart muscle (*n* = 7) and/or fibroblast cell line (*n* = 26).

### Molecular genetic investigations

**Fig. 1:**
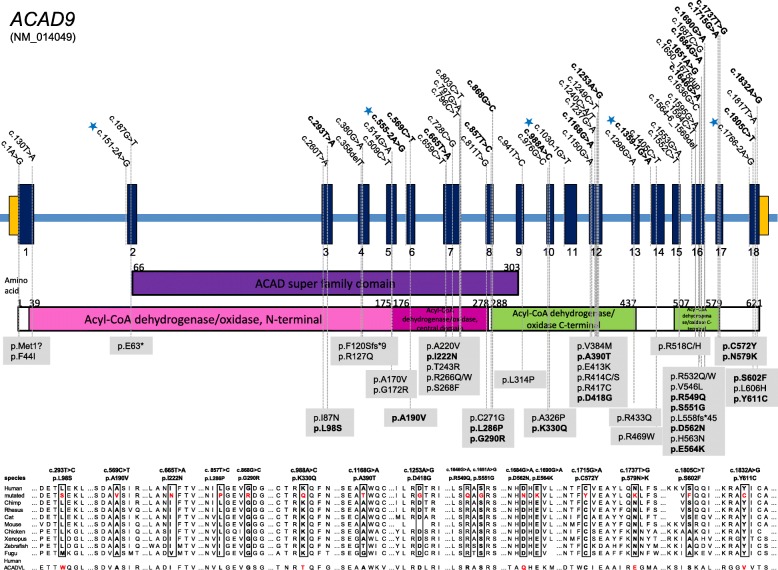
*ACAD9* mutation status, gene structure and conservation of affected amino acid residues. Gene structure of ACAD9 with localization of mutations in 70 patients. Blue asterisks indicate splice site mutations. Newly identified mutations are shown in bold. Conservation of amino acid residues affected by missense variants

Figure 1 and Additional file 2: Table S1 present the 18 previously unreported variants (in bold) and 34 known variants in *ACAD9* found in our cohort. Of these 42 were missense, one frame shift, one nonsense, seven splice site and one start codon mutation. No individual harbored two variants predicted to lead to a loss of protein function.

Based on the prevalence of deleterious *ACAD9* alleles in the normal population (GnomAD, www.gnomad.broadinstitute.org, [33]) we estimated that approximately 59 children with ACAD9 deficiency will be born each year in Europe (for calculation see Additional file 3 Table S2).

### Clinical spectrum

**Fig. 2:**
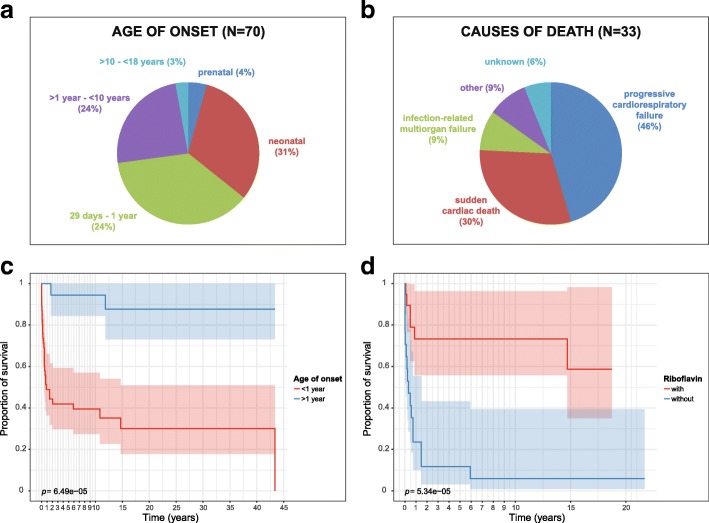
Age of onset, causes of death, survival and effect of riboflavin on survival of ACAD9 patients. **a** Age of onset of symptoms, (**b**) Causes of death, (**c**) Kaplan-Maier survival rates. In red, patients with a disease presentation in the first year of life. In blue, patients with a later presentation (*p* = 6.49e-05). **b** In red, patients with a disease presentation in the first year of life and treated with riboflavin. In blue, patients of the same age category but untreated with riboflavin (*p* = 5.34e-05, confidence 95%)

The data are summarized in Table 1 and Additional file 4: Table S3. Not all data were available for all patients, the denominator indicates the number of patients for which data were available. Currently 37 individuals are alive at a median age of 14 years (range 24 days – 44 years), the median age of patients deceased was 3 months (range 1 day – 44 years). Patients with a presentation in the first year of life (*n* = 50) show a significantly worse survival when compared to patients presenting later (*n* = 20, Fig. 2a). One individual (I18) was reported with fetal cardiomegaly, two were reported with fetal rhythm abnormalities, all passed away early, on day 1 (I18), 2 (I42) and 280 (I55), respectively.

**Table 1:** Main clinical findings

	Number	n available	Percent
Prenatal findings			
Cardiomegaly	1	60	2
Rhythm abnormalities	2	60	3
Decreased child movements	1	60	2
Oligohydramnios	4	60	7
Intrauterine growth failure	6	60	10
Neonatal course			
Lactic acidosis	21	63	33
Cardiomyopathy	15	63	24
Rhythm abnormalities	4	54	7
Respiratory failure necessitating artificial ventilation	6	54	11
Severe liver dysfunction/failure	2	54	4
Severe renal dysfuntion/failure	2	54	4
Most frequent clinical findings			
Cardiomyopathy at presentation/during course	44/56	66/66	67/85
Muscular weakness at presentation/during course	21/37	48/49	44/75
Exercise intolerance at presentation/during course	21/34	49/47	43/72
Neurological findings			
Severe intellectual disability (clinical impression)	1	51	2
Mild intellectual disability (clinical impression)	14	48	29
Severe developmental delay (clinical impression)	4	52	8
Mild developmental delay (clinical impression)	23	51	45
Optic atrophy, retinits pigmentosa	0	70	0
Neuroradiological findings			
MRI: basal ganglia alterations	4	24	17
MRI: leukoencephalopathy	5	21	24
MRI: global brain atrophy	2	20	10
MRI: isolated cerebellar atrophy	1	20	5
MRS: lactate peak (any location)	2	16	13
Activities of daily living			
Age adequate behaviour	27	39	69
Attending/finished regular school	26	41	63
Able to sit independently	34	42	81
Able to walk independently	33	41	80
Able to eat and drink independently	33	41	80
Able to perform personal hygiene independently	30	41	73
Able to communicate with words/sentences	34/31	43/42	79/74

### Cardiomyopathy and treatment

I20 presented with hypertrophic cardiomyopathy in the first year of life. Due to rapid deterioration, she received heart transplantation at 2 years. She died of cardiac failure 4 years later. I21 presented with hypertrophic cardiomyopathy at 18 months and subsequently developed neurological symptoms (ataxia and epilepsy), which were non-progressive and mild. She was successfully heart transplanted at the age of 9 years and is currently 15 years old. I22, currently 35 years old, presented with a progressive biventricular hypertrophic cardiomyopathy in childhood and was transplanted at the age of 18 years. After a follow up of six and 17 years, respectively, their cardiac function remained satisfactory. I30 showed tachycardia in the first days of life birth and signs of heart failure at 1 month. Despite undergoing cardioverter-defibrillator implantation and subsequent heart transplantation, he died at 3 months of age.

Regarding drug treatment, a positive effect on heart failure was reported for beta-blocking agents (14/44 = 32%), ACE inhibitors (6/40 = 15%), calcium-channel blockers (1/37 = 3%) and diuretics (3/39 = 8%). No patient received digitoxin or digoxin. A worsening effect was only reported for one patient on beta blockers.

### Riboflavin and other oral vitamin treatment

Of the entire cohort of 67 patients, 20 patients were reported as not treated; data about treatment and/or effect were unavailable for 15 patients. Data on the general clinical effect of riboflavin as reported by the responsible physician were available for 31 patients. For 20 patients (20/31 = 65%) physicians reported a beneficial effect, for 11 (35%) no effect. No clinical deterioration or side effects were reported. Detailed data on onset of riboflavin treatment, dosage, duration and clinical effect were only available for a minority of patients and have not been investigated.

To analyze the effect of riboflavin treatment, we focused on the patients presenting during the first year of life as these was the biggest subgroup and the group with the shortest survival suggesting the most severe course. For 39 of these 50 patients, data on riboflavin treatment were available (*n* = 17 untreated, *n* = 22 treated). Figure 2b shows the Kaplan-Meier curve for both groups of patients and indicates a significantly better survival rate for patients with oral riboflavin treatment (deceased *n* = 7/22) in contrast to untreated patients (deceased *n* = 16/17).

Regarding other food supplements, several patients were reported as taking coenzyme Q10, biotin and L-carnitine with anecdotal positive effects.

### Cell culture experiments

#### Effect of different ACAD9 mutations on ACAD9 protein level and respiratory chain complex I activity

**Fig. 3:**
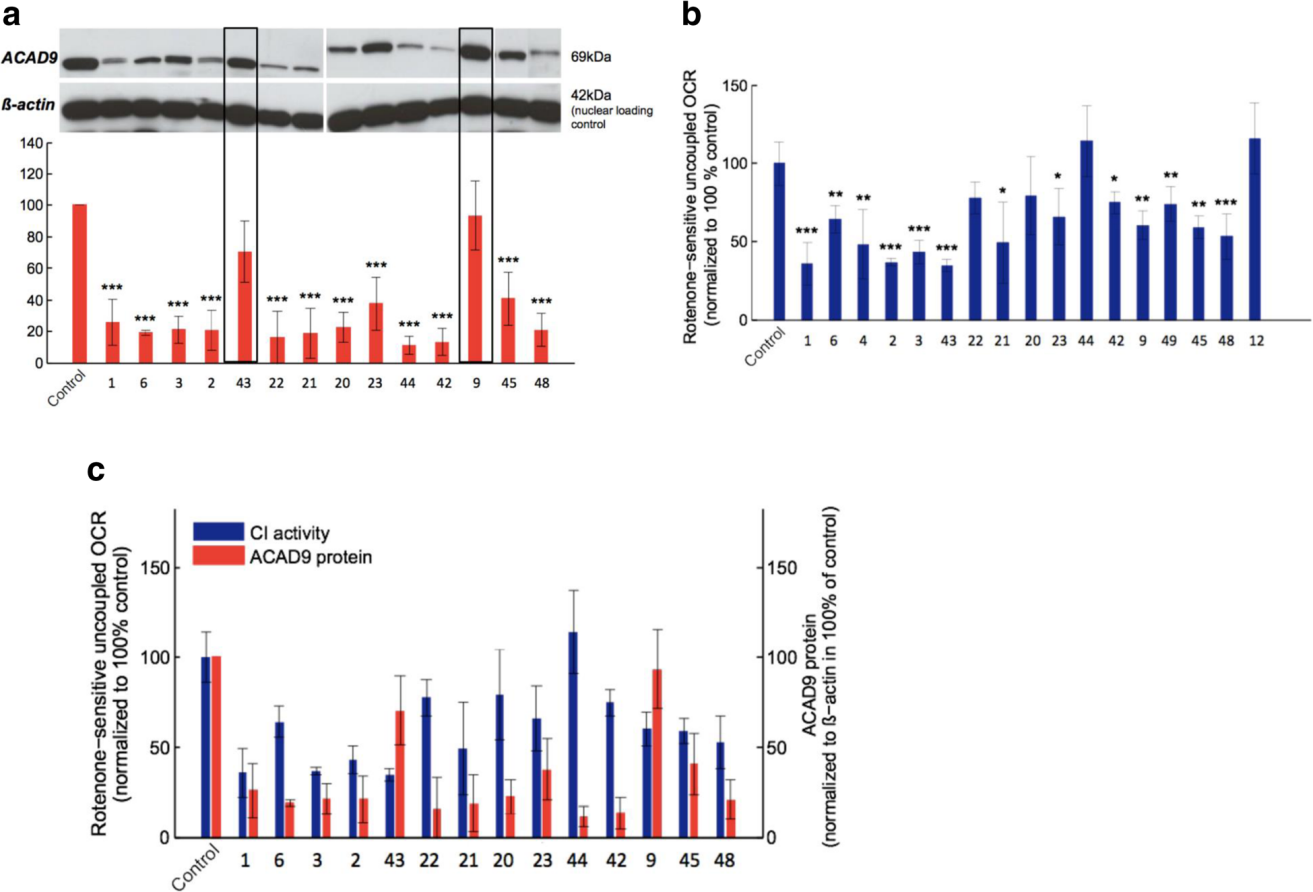
Measurements of ACAD9 protein level and complex I activity in patient derived fibroblasts. **a** Western blot and quantification of ACAD9 protein levels in patient derived fibroblasts and control. **b** Complex I activity in patient derived fibroblasts and control. **c** Comparison between remaining ACAD9 protein (red) and Complex I activity (blue). Data expressed as average of three independent western blots and average of > 10 technical replicates (oxygen consumption rate ± SD)

ACAD9 levels were significantly reduced in all but two of 14 examined patient fibroblast cell lines; both exceptional cell lines (F9, F43) carried a homozygous p.(Arg518His) variant and showed normal ACAD9 levels (Fig. 3a).

Complex I-dependent respiration was found to be significantly decreased in 13 of 17 evaluated patient cell lines. The cell lines F44 (homozygous p.[Leu98Ser]) and F12 (homozygous p.[Arg532Trp]) showed no complex I deficiency; F22 (p.[splice];[Arg433Gln]) and F20 (p.[Phe120Serfs*9];[Arg532Trp] showed only mildly reduced levels (Fig. 3b).

There was no correlation between complex I activity and residual ACAD9 protein levels. Interestingly, nearly normal complex I activity was recorded in the cell line of F42 (p.[Glu564Lys];[Tyr611Cys]) despite very low steady-state ACAD9 protein levels, indicating that the remaining ACAD9 chaperone activity might be high enough to correct assembly of complex I (Fig. 3c).

#### Respiratory chain complex I activity after bezafibrate and riboflavin supplementation

**Fig. 4:**
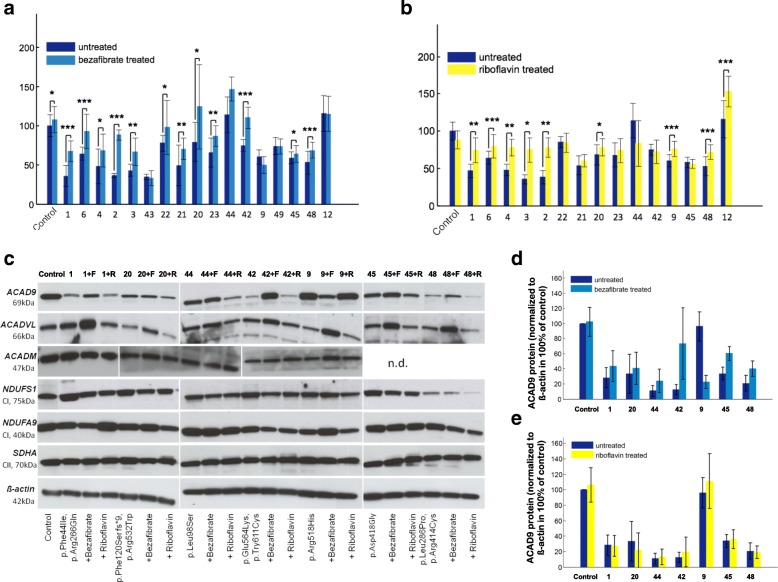
Effect of bezafibrate and riboflavin supplementation on respiratory chain activities in fibroblast cell lines. Maximal oxygen consumption rate (OCR) was measured in pmol/(s*Mill) of *ACAD9* patient and control fibroblasts with and without (**a**) bezafibrate (400 μM for 72 h) and (**b**) riboflavin (530 nM for 72 h) treatment. Data are expressed as the average of > 10 technical replicates and normalized to control. ± SD. ***P < 0.001, **P < 0.01, *P < 0.05. (**c**) Whole cell lysate of control and different *ACAD9* deficient fibroblasts +/− bezafibrate/riboflavin visualized with antibodies against ACAD9, ACADVL, ACADM, SDHA, ß-actin (loading control), NDUFS1 and NDUFA9 (**d**, **e**) Quantification of ACAD9 and ACADVL protein levels

After 72 h of bezafibrate treatment, the complex I activity increased in the control and in all but three patients cell lines. The increase was significant in 12 out of 17 patient cell lines. Five patient cell lines reached almost normal levels (Fig. 4a). The already normal complex I activity of two cell lines (**F44 and F12**) remained unchanged.

Riboflavin supplementation led to a significant improvement of complex I activity in nine out of 15 patient cell lines and had no effect in the remaining six cell lines. The increase ranged from 14 to 109% (Fig. 4b).

#### Supercomplex formation after bezafibrate treatment

All investigated cell lines had a clear reduction of assembled supercomplexes, however, there was no correlation between the amount of ACAD9 protein and the extent of supercomplex formation, (e.g. F23 with almost normal amount of ACAD9 presented a complete loss of supercomplexes whereas F44 with nearly absent ACAD9 protein presented a high amount of assembled supercomplexes). This indicates that small quantities of productive ACAD9 can fulfill assembly function. An increase in the assembly of supercomplexes was found in four out of five cell lines treated with bezafibrate (Additional file 5 Figure S1).

#### Findings in cell culture versus clinical effect in patients supplemented with riboflavin

The same mutation as in I12/F12 has previously been reported in I6/F6 (P2 in [7]). I6 was reported to benefit from oral riboflavin. Both cell lines, F6 and F12, consequently showed improving complex I activity under riboflavin treatment (Fig. 3b). Data for treatment of paired cells and patients were only available for eight patients. In seven pairs the effect was concordant in cells and patients. Six pairs (I/F2, 3, 4, 6, 9, 12) showed positive effects both in cell culture and clinical, one pair (I/F44) did not show any response. I48 did clinically not respond to treatment, whereas his cells did.

## Discussion

Complex I deficiency is the most common biochemical signature of mitochondrial disorders. Given the number of ACAD9 deficient patients described here for a disorder genetically defined only in 2010, and based on the frequency of deleterious alleles described to date, ACAD9 is likely to be one of the more common causes of mitochondrial respiratory chain deficiency, with a conservative estimate of 59 new patients born every year in Europe, and 689 world-wide (Additional file 3 Table S2).

The mutations of the 70 patients from 50 families with ACAD9 deficiency were located across the coding sequence of the gene, with no founder mutations identified. However, no individual harbored two clear loss of function alleles, suggesting that a complete loss of ACAD9 function might be incompatible with life. This is also supported by the fact that the homozygous knock out mouse was found to be embryonic lethal (Schiff, Vockley, *personal communication*). No genotype-phenotype correlation for mutations could be identified based on specific regions of the gene or functional domains of the protein.

The vast majority of patients presented with hypertrophic cardiomyopathy, lactic acidosis, muscle weakness, and exercise intolerance. However, patients without cardiomyopathy also were identified our study. Although both ACAD9 and VLCAD deficiency can present with cardiomyopathy, the clinical phenotype is otherwise distinct, with hypoglycemia, rhabdomyolysis and liver failure, typically seen in VLCAD. These symptoms were infrequently seen in our ACAD9 deficient cohort.

Our data suggest, that there are two subgroups of ACAD9 deficient patients. Patients who presented in the first year of life often died early and, if surviving, did more poorly than those who presented later. In contrast to many other mitochondrial disorders, severe intellectual disability and developmental delay, as well as other neurological features, were seen in only a minority of (surviving) patients. Indeed, all patients with severe developmental delay (*n* = 4) or intellectual delay (*n* = 1) had early disease onset. Furthermore, most patients currently alive were able to perform routine activities of daily living.

This observation, is not only very important for providing anticipatory guidance, but might also influence a decision regarding heart transplantation. Four patients of our cohort underwent heart transplantation. Unfortunately, the two patients who presented within the first year died despite all efforts. In contrast, the two patients presenting after the age of 1 year developed normally and are currently aged 15 and 35 years, respectively. Additional longitudinal studies are warranted to better identify which patients with ACAD9 deficiency are appropriate heart transplant candidates.

Supplementation with riboflavin showed improvement in complex I activity in the vast majority of patient fibroblasts, and most patients similarly were reported to have clinical benefit with treatment. Most notably, patients presenting within the first year of life show a significant better survival when treated with riboflavin. One limitation of this observation could be that most of the deaths occurred at the end of the first year of life. This might indicate that our analysis is prone to survivor treatment selection bias. Detailed data about the starting point of riboflavin treatment, the dosage etc. in more patients are needed.

This observation supports anecdotal reports in the literature. In our cohort, families 1 and 33 are particularly instructive. In both families the first child (I1, I45) died within the first 2 years of life without riboflavin supplementation, whereas the younger affected siblings (I2, I45 and I46), in whom supplementation was begun immediately upon diagnosis, are currently still alive (aged 10, 1.5 and 11 years, retrospectively). Cases I5 and I6 were first reported with riboflavin responsive complex I deficiency before their molecular defect was known [7, 34]. Paired data on fibroblasts and patient riboflavin treatment were available for eight patients, six of which showed parallel beneficial effects and one no effect. Further cellular studies are necessary to define the mode of action of riboflavin in ACAD9 deficiency.

The PPAR promotor activator bezafibrate has been reported to be of benefit in other FAODs. In all cell lines examined in this study, bezafibrate improved the formation of respiratory chain super complexes, likely explaining the improved respiration of the patient cell lines as measured by whole cell oximetry. While only a limited number of cell lines were tested, these results suggest a potential role for bezafibrate or other PPAR activators in the treatment of ACAD9. However, similar effects for bezafibrate have been reported in cell models of other fatty acid oxidation defects, but were not proven in humans.

Our retrospective data provides additional description of the clinical and genetic spectrum of ACAD9 deficiency, and provides valuable insight for the development of future clinical trials of riboflavin, bezafibrate, or other therapies. While the current study was not designed to be a clinical trial, the anecdotal improvement of many ACAD9 deficient patients to riboflavin justifies a trial of riboflavin (20 mg/kg/day, maximum 200 mg/day) in every patient with this diagnosis. Given the high frequency of ACAD9 deficiency, we propose that it would be reasonable to consider riboflavin administration for phenotypically-consistent patients whilst their genetic investigations are underway [35]. This also underlines that in patients with suspicion of a mitochondrial disorder next generation sequencing techniques should be initiated promptly, in selected cases accompanied by studies in affected tissues. For these patients, early diagnosis and therapeutic intervention could be the difference between life and death.

## Conclusions

ACAD9 typically presents with cardiomyopathy, exercise intolerance and muscular weakness and the clinical course might respond to riboflavin.

## Additional files

Additional file 1: Additional data. (DOCX 38 kb)

Additional file 2: **Table S1.** Compound heterozygous and homozygous *ACAD9* variants identified in 67 patients present in this study (DOCX 75 kb)

Additional file 3: **Table S2.** Calculation of European incidence of ACAD9 deficiency (DOCX 36 kb)

Additional file 4: **Table S3.** Clinical characteristics of the 67 patients present in this study (DOCX 72 kb)

Additional file 5: **Figure S1.** Representative picture of Complex I assembly in fibroblasts of individual 1 (A, upper panels) Two-dimensional BN/SDS-PAGE separation and quantification of fluorescent-labelled mitochondrial complexes from 10 mg patient (left) and control fibroblasts (right) are shown. (A, lower panels) show silver stained 2 D gels. (B) Quantified Supercomplexes in 2D gels from control and patient fibroblast with and without bezafibrate treatment for 72 h. (C) Panoramaplots of 2D gels with assignment of signals used for quantification of complexes. Assignment of complexes: O, OGDC, oxoglutarate dehydrogenase complex; V, complex V or ATP synthase; III, complex III or cytochrome c reductase; IV, complex IV or cytochrome c oxidase; S, supercomplexes composed of respiratory chain complexes I, III, and IV. 2-D gels were scanned side by side for direct comparison and are shown as pseudocolors. (pdf). (PDF 1109 kb)

## Abbreviations

ACAD: Acyl-CoA dehydrogenase
F: Patient derived fibroblast cell line
FAO: Fatty acid oxidation
I: Individual
OCR: Oxygen consumption rate
OXPHOS: Oxidative phosphorlyation system
VLCAD: Very long-chain acyl-CoA dehydrogenase
