# Supplementary material for: Clinical, biochemical and genetic spectrum of 70 patients with ACAD9 deficiency: is riboflavin supplementation effective?
Source: Orphanet J Rare Dis. 2018 Jul 19;13:120. doi: 10.1186/s13023-018-0784-8 (PMC6053715; doi:10.1186/s13023-018-0784-8)
Supplement: Supplementary file 1 — Additional data. (DOCX 38 kb) [file 13023_2018_784_MOESM1_ESM.docx]

**SUPPLEMENTARY DATA**

*Electrophoresis and in-gel quantification of fluorescent-labeled proteins*

Standard protocols for solubilization of cells with digitonin, 1-D BNE (blue native electrophoresis), and 2-D SDS-PAGE [31] [32] were modified to improve isolation of mitochondrial complexes from human fibroblasts. Briefly, mitochondrial protein complexes from 10 mg fibroblasts (wet weight) were labeled with Fluorescein and solubilized with 5µl digitonin (20%) in a buffer containing 50 mM NaCl, 50 mM imidazole, 2 mM aminohexanoic acid, and 1mM EDTA, pH 7. Following 1-D BNE and 2-D SDS-PAGE, the 2-D gels were scanned using a Typhoon scanner (GE Healthcare) to detect Fluorescein (excitation 488nm, emission filter 520nm). Quantity One software (BioRad) was used for densitometric quantification of the fluorescence intensities in 2-D gels. A few clearly visible signals were selected for quantification (supplementary figure 1C, a double band comprising subunits OSCP and d of ATP synthase, a double band comprising Cox4a,b of complex IV, a band assigned to respiratory supercomplexes containing the 49kDa (NDUFS2) and 51kDa (NDUFV1) proteins of complex I and the core subunits of complex III.

**Legend** **Supplementary Figure S-1: Representative picture of Complex I assembly in fibroblasts of individual 1**

(A, upper panels) Two-dimensional BN/SDS-PAGE separation and quantification of fluorescent-labelled mitochondrial complexes from 10mg patient (left) and control fibroblasts (right) are shown. (A, lower panels) show silver stained 2 D gels. (B) Quantified Supercomplexes in 2D gels from control and patient fibroblast with and without bezafibrate treatment for 72h. (C) Panoramaplots of 2D gels with assignment of signals used for quantification of complexes. Assignment of complexes: O, OGDC, oxoglutarate dehydrogenase complex; V, complex V or ATP synthase; III, complex III or cytochrome c reductase; IV, complex IV or cytochrome c oxidase; S, supercomplexes composed of respiratory chain complexes I, III, and IV. 2-D gels were scanned side by side for direct comparison and are shown as pseudocolors.
