# Supplementary material for: Clinical, biochemical and genetic spectrum of 70 patients with ACAD9 deficiency: is riboflavin supplementation effective?
Source: Orphanet J Rare Dis. 2018 Jul 19;13:120. doi: 10.1186/s13023-018-0784-8 (PMC6053715; doi:10.1186/s13023-018-0784-8)
Supplement: Supplementary file 2 — Table S1. Compound heterozygous and homozygous ACAD9 variants identified in 67 patients present in this study (DOCX 75 kb) [file 13023_2018_784_MOESM2_ESM.docx]

**Table S1**

| **ID,**  **family,**  **sex** | **Country** | **Ref.** | **cDNA** | **Protein** | **Variant type** | **MAF** | **cDNA** | **Protein** | **Variant**  **type** | **MAF** |
| --- | --- | --- | --- | --- | --- | --- | --- | --- | --- | --- |
| 1, 1, F | Italy | Pat.1, #35834, [5] | c.130T>A | p.Phe44Ile | missense | np | c.797G>A | p.Arg266Gln | missense | 1.219e-5 |
| 2, 1, M | Italy | Pat.2, #52935, [5] | c.130T>A | p.Phe44Ile | missense | np | c.797G>A | p.Arg266Gln | missense | 1.219e-5 |
| 3, 2, F | Italy | Pat.3, #52933, [5] | c.797G>A | p.Arg266Gln | missense | 1.219e-5 | c.1249C>T | p.Arg417Cys | missense | np |
| 4, 3, F | UK | Pat.4, #52674, [5] | c.976G>C | p.Ala326Pro | missense | 5.279e-5 | c.1594C>T | p.Arg532Trp | missense | 4.089e-6 |
| 5, 4, F | Netherlands | Pat.1, CB, VII:11, [7] | c.1594C>T | p.Arg532Trp | missense | 4.089e-6 | c.1594C>T | p.Arg532Trp | missense | 4.089e-6 |
| 6, 4, F | Netherlands | Pat.2, #49591, MJ, VII:6, [7] | c.1594C>T | p.Arg532Trp | missense | 4.089e-6 | c.1594C>T | p.Arg532Trp | missense | 4.089e-6 |
| 7, 4, M | Netherlands | Pat.3, JJ, VII:8, [7] | c.1594C>T | p.Arg532Trp | missense | 4.089e-6 | c.1594C>T | p.Arg532Trp | missense | 4.089e-6 |
| 8, 5, F | Netherlands | Pat.4, CV, [7] | c.380G>A | p.Arg127Gln | missense | np | c.1405C>T | p.Arg469Trp | missense | 0.0003754 |
| 9, 6, F | Canada | Pat.1, #69842, [6] | c.1553G>A | p.Arg518His | missense | 8.66e-5 | c.1553G>A | p.Arg518His | missense | 8.66e-5 |
| 10, 7, F | Thailand/  Germany | Pat.2 (twin sister), [6] | c.187G>T | p.Glu63X | nonsense | np | c.1237G>A | p.Glu413Lys | missense | 1.625e-5 |
| 11, 7, F | Thailand/  Germany | Pat.3 (twin sister), [6] | c.187G>T | p.Glu63X | nonsense | np | c.1237G>A | p.Glu413Lys | missense | 1.625e-5 |
| 12, 8, M | Turkey | Pat.1, #59029, 72545, [12] | c.1594C>T | p.Arg532Trp | missense | 4.089e-6 | c.1594C>T | p.Arg532Trp | missense | 4.089e-6 |
| 13, 8, F | Turkey | Pat.2, #59033, [12] | c.1594C>T | p.Arg532Trp | missense | 4.089e-6 | c.1594C>T | p.Arg532Trp | missense | 4.089e-6 |
| 14, 8, F | Turkey | Pat.3, #59036, [12] | c.1594C>T | p.Arg532Trp | missense | 4.089e-6 | c.1594C>T | p.Arg532Trp | missense | 4.089e-6 |
| 15, 9, M | Not available | [33] | c.260T>A | p.Ile87Asn | missense | np | c.976G>A | p.Ala326Pro | missense | np |
| 16, 10, M | Italy | [13] | c.1240C>T | p.Arg414Cys | missense | 1.219e-5 | c.1240C>T | p.Arg414Cys | missense | 1.219e-5 |
| 17, 11, F | Turkey | [34] | c.659C>T | p.Ala220Val | missense | np | c.659C>T | p.Ala220Val | missense | np |
| 18, 12, M | France | [15] | c.1030-1G>T | acceptor splice | splice | np | c.1249C>T | p.Arg417Cys | missense | np |
| 19, 13, F | West Africa | P2, [18] | c.976G>A | p.Ala326Pro | missense | 5.279e-5 | c.1595G>A | p.Arg532Gln | missense | 8.174e-6 |
| 20, 14, F | Portugal | P3, [18] | c.358delT | p.Phe120fs | frameshift | 0.000109 | c.1594C>T | p.Arg532Trp | missense | 4.089e-6 |
| 21, 15, F | France | P4, [18] | c.976G>C | p.Ala326Pro | missense | 5.279e-5 | c.1595G>A | p.Arg532Gln | missense | 8.174e-6 |
| 22, 16, F | France | P5, [18] | c.151-2A>G | acceptor splice | splice site | np | c.1298G>A | p.Arg433Gln | missense | 4.062e-6 |
| 23, 17, M | French Caribbean | P6, [18] | c.1237G>A | p.Glu413Lys | missense | 1.625e-5 | c.1552C>T | p.Arg518Cys | missense | 8.66e-5 |
| 24, 18, F | French Caribbean | P7, [18] | c.1552C>T | p.Arg518Cys | missense | 8.66e-5 | c.1564-6_1569del | splice site | splice | np |
| 25, 19, F | French Caribbean | P8, [18] | c.1A>G | p.Met1? | start lost | 1.227e-5 | c.796C>T | P.Arg266Trp | missense | 8.123e-6 |
| 26, 20, F | Japan | Pt090, [19] | c.1150G>A | p.Val384Met | missense | 4.065e-6 | c.1817T>A | p.Leu606His | missense | np |
| 27, 21, F | Japan | Pt025, [19] | c.811T>G | p.Cys271Gly | missense | np | c.1766-2A>G | splice site | splice site | np |
| 28, 22, M | not available | [21] | c.187G>T | p.Glu63X | nonsense | np | c.941T>C | p.Leu314Pro | missense | np |
| 29, 23, F | Poland | P1, [22] | c.514G>A | p.Gly172Arg | missense | 1.804e-5 | c.803C>T | p.Ser268Phe | missense | 8.124e-6 |
| 30, 24, M | Poland | P2, [22] | c.1552C>T | p.Arg518Cys | missense | 8.66e-5 | c.1553G>A | p.Arg518His | missense |  |
| 31, 25, M | Poland | P3, [22] | c.728C>G | p.Thr243Arg | missense | 8.121e-6 | c.1552C>T | p.Arg518Cys | missense | 8.66e-5 |
| 32, 26, F | Morocco | I-1, [8] | c.1636G>C | p.Val546Leu | missense | np | c.1636G>C | p.Val546Leu | missense | np |
| 33, 26, F | Morocco | I-2, [8] | c.1636G>C | p.Val546Leu | missense | np | c.1636G>C | p.Val546Leu | missense | np |
| 34, 26, M | Morocco | I-3, [8] | c.1636G>C | p.Val546Leu | missense | np | c.1636G>C | p.Val546Leu | missense | np |
| 35, 26, F | Morocco | I,4, [8] | c.1636G>C | p.Val546Leu | missense | np | c.1636G>C | p.Val546Leu | missense | np |
| 36, 27, M | Belgium | II-1, [8] | c.509C>T | p.Ala170Val | missense | 1.218e-5 | c.1687C>G | p.His563Asp | missense | np |
| 37, 27, F | Belgium | II-2, [8] | c.509C>T | p.Ala170Val | missense | 1.218e-5 | c.1687C>G | p.His563Asp | missense | np |
| 38, 28, F | Congo | III-3, [8] | c.1240C>A | p.Arg414Ser | missense | 4.062e-6 | c.1650_1672dup | p.Leu558fs | frameshift | np |
| 39, 28, F | Congo | III-6, [8] | c.1240C>A | p.Arg414Ser | missense | 4.062e-6 | c.1650_1672dup | p.Leu558fs | frameshift | np |
| 40, 28, F | Congo | III-7, [8] | c.1240C>A | p.Arg414Ser | missense | 4.062e-6 | c.1650_1672dup | p.Leu558fs | frameshift | np |
| 41, 29, F | Tunisia | [35] | c.1240C>T | p.Arg414Cys | missense | 1.219e-5 | c.1240C>T | p.Arg414Cys | missense | 1.219e-5 |
| 42, 30, M | Austria | This paper | c.1690G>A | p.Glu564Lys | missense | 4.074e-6 | c.1832A>G | p.Tyr611Cys | missense | 8.121e-6 |
| 43, 31, M | Pakistan | This paper | c.1553G>A | p.Arg518His | missense | 1.083e-5 | c.1553G>A | p.Arg518His | missense | 1.083e-5 |
| 44, 32, F | Asia | This paper | c.293T>C | p.Leu98Ser | missense | np | c.293T>C | p.Leu98Ser | missense | np |
| 45, 33, F | Sri Lanka | This paper | c.1253A>G | p.Asp418Gly | missense | np | c.1253A>G | p.Asp418Gly | missense | np |
| 46, 33, M | Sri Lanka | This paper | c.1253A>G | p.Asp418Gly | missense | np | c.1253A>G | p.Asp418Gly | missense | np |
| 47, 33, M | Sri Lanka | This paper | c.1253A>G | p.Asp418Gly | missense | np | c.1253A>G | p.Asp418Gly | missense | np |
| 48, 34, F | Italy | This paper | c.857T>C | p.Leu286Pro | missense | np | c.1240C>T | p.Arg414Cys | missense | 1.219e-5 |
| 49, 35, F | France | This paper | c.976G>C | p.Ala326Pro | missense | 5.279e-5 | c.1651A>G | p.Ser551Gly | missense | np |
| 50, 36, M | Bahrain | This paper | c.1684G>A | p.Asp562Asn | missense | 4.34e-5 | c.1684G>A | p.Asp562Asn | missense | 4.34e-5 |
| 51, 37, F | not available | This paper | c.1805C>T | p.Ser602Phe | missense | np | c.1805C>T | p.Ser602Phe | missense | np |
| 52, 37, M | not available | This paper | c.1805C>T | p.Ser602Phe | missense | np | c.1805C>T | p.Ser602Phe | missense | np |
| 53, 38, M | Nigeria | This paper | c.868G>A | p.Gly290Arg | missense | 3.228e-5 | c.1237G>A | p.Glu413Lys | missense | 1.625e-5 |
| 54, 38, M | Nigeria | This paper | c.868G>A | p.Gly290Arg | missense | 3.228e-5 | c.1237G>A | p.Glu413Lys | missense | 1.625e-5 |
| 55, 38, M | Nigeria | This paper | c.868G>A | p.Gly290Arg | missense | 3.228e-5 | c.1237G>A | p.Glu413Lys | missense | 1.625e-5 |
| 56, 39, F | UK | This paper | c.976G>C | p.Ala326Pro | missense | 5.279e-5 | c.1594C>T | p.Arg532Trp | missense | 4.089e-6 |
| 57, 40, M | UK | This paper | c.665T>A | p.Ile222Asn | missense | np | c.1249C>T | p.Arg417Cys | missense | np |
| 58, 40, M | UK | This paper | c.665T>A | p.Ile222Asn | missense | np | c.1249C>T | p.Arg417Cys | missense | np |
| 59, 41, M | UK | This paper | c.1150G>A | p.Val384Met | missense | 4.065e-6 | c.1168G>A | p.Ala390Thr | missense | 1.444e-5 |
| 60, 41, M | UK | This paper | c.1150G>A | p.Val384Met | missense | 4.065e-6 | c.1168G>A | p.Ala390Thr | missense | 1.444e-5 |
| 61, 42, F | UK | This paper | c.1552C>T | p.Arg518Cys | missense | 8.66e-5 | c.1715G>A | p.Cys572Tyr | missense | np |
| 62, 43, F | Belgium | This paper | c.976G>C | p.Ala326Pro | missense | 5.279e-5 | c.1552C>T | p.Arg518Cys | missense | 8.66e-5 |
| 63, 44, M | Italy | This paper | c.1240C>T | p.Arg414Cys | missense | 1.219e-5 | c.1646G>A | p.Arg549Gln | missense | 8.18e-6 |
| 64, 45, F | Germany/  Poland | This paper | c.569C>T | p.Ala190Val | missense | 4.074e-6 | c.1405C>T | p.Arg469Trp | missense | 0.0003754 |
| 65, 46, M | not available | This paper | c.1240C>T | p.Arg414Cys | missense | 1.219e-5 | c.1240C>T | p.Arg414Cys | missense | 1.219e-5 |
| 66, 46, M | not available | This paper | c.1240C>T | p.Arg414Cys | missense | 1.219e-5 | c.1240C>T | p.Arg414Cys | missense | 1.219e-5 |
| 67, 47, M | not available | This paper | c.555-2A>G | splice site | splice site | np | c.1168G>A | p.Ala390Thr | missense | 1.444e-5 |
| 68, 48, M | China | This paper | c.797G>A | p.Arg266Gln | missense | 1.219e-5 | c.1359-1G>A | splice site | splice site | np |
| 69, 49, F | China | This paper | c.1737T>G | p.Asn579Lys | missense | np | c.1240C>T | p.Arg414Cys | missense | 1.219e-5 |
| 70, 50, F | China | This paper | c.988A>C | p.Lys330Gln | missense | 0.002096 | c.988A>C | p.Lys330Gln | missense | 0.002096* |

MAF=Minor allele frequency reported in gnomAD, np=not present,* uncertain diagnosis due to high allele frequency
