# Supplementary material for: Clinical, biochemical and genetic spectrum of 70 patients with ACAD9 deficiency: is riboflavin supplementation effective?
Source: Orphanet J Rare Dis. 2018 Jul 19;13:120. doi: 10.1186/s13023-018-0784-8 (PMC6053715; doi:10.1186/s13023-018-0784-8)
Supplement: Supplementary file 3 — Table S2. Calculation of European incidence of ACAD9 deficiency (DOCX 36 kb) [file 13023_2018_784_MOESM3_ESM.docx]

**Table S2.** Calculation of European and worldwide incidence of ACAD9 deficiency

|  |  | Formula |  |  |
| --- | --- | --- | --- | --- |
| *ACAD9* LoFs^*^ in GnomAD** | (A) | Allele count – n homozygous carrier/  1/2* allele number | European 0,000666345  Total 0,000689169 |  |
| Causal ACAD9 missense and LoF variants (this paper) | (B) | Allele count- n homozygous carrier/  1/2* allele number | European 0,002762796  Total 0,001618123 |  |
| Total allele frequency (missense + LoF) in GnomAD and this paper | (C) | (A+B)^2^ | European 0,00001176  Total 0,0000053236 |  |
| Estimated prevalence | (D) | 1/C | European 1:85041  Total 1:187842 |  |
| Estimated incidence in Europe^#^  Worldwide extrapolation § | (E)  (F) | 5.1*10^6^/D  129.596 *10^6^/D | 59  689 |  |

^*^LoF: unequivocal, splice site, and frameshift variants in the canonical *ACAD9* transcript^#^ European population: 5.1 million births per year. Worldwide population: 129.596 million births per year. § under the assumption of the same allele frequency worldwide which is likely not the case. ^**^approximately 250,000 *ACAD9* alleles were tested.

**Supplementary table 2.** To estimate the incidence of ACAD9 deficiency in the European population we calculate the minor allele frequency (Supp. Table 2- A) of LOF variants contained in GnomAD and the causal ACAD9 variants present in this paper, using European (not Finnish) MAF for the European calculation and the total MAF for the worldwide calculation. Then we used the total allele frequency (Supp. table 2- C) to estimate the prevalence of ACAD9 deficiency (Supp. table 2- D). The incidence was then calculated in the European population (5.1 millions of individuals) and then a worldwide extrapolation was performed with the important limitation based on allele frequency data.
