# Supplementary material for: Clinical, biochemical and genetic spectrum of 70 patients with ACAD9 deficiency: is riboflavin supplementation effective?
Source: Orphanet J Rare Dis. 2018 Jul 19;13:120. doi: 10.1186/s13023-018-0784-8 (PMC6053715; doi:10.1186/s13023-018-0784-8)
Supplement: Supplementary file 4 — Table S3. Clinical characteristics of the 67 patients present in this study (DOCX 72 kb) [file 13023_2018_784_MOESM4_ESM.docx]

**Table S3**

| **ID, Family, Sex** | **Ref.** | **Age of onset** | **Age of onset*** | **Current age in day (* if dead)** | **Prenatal**  **findings** | **Cardio-myopathy** | **Arrhythmias,**  **any kind** | **Cardiological**  **features** | **Neurological**  **features** | **Effect of Riboflavin (rated by physician)** | **Effect of Riboflavin (cells)** |
| --- | --- | --- | --- | --- | --- | --- | --- | --- | --- | --- | --- |
| 1, 1, F | Pat.1, #35834, [5] | neonatal | 1 | 49* |  | PS | NA | HCM | NA | untreated | beneficial effect |
| 2, 1, M | Pat.2, #52935, [5] | neonatal | 1 | 3600 |  | PS | NA | HCM | no | beneficial effect | beneficial effect |
| 3, 2, F | Pat.3, #52933, [5] | early infancy | 2 | 4320* |  | PS | NA | HCM | no | beneficial effect | beneficial effect |
| 4, 3, F | Pat.4, #52674, [5] | 1 w | 1 | 750* |  | DS | no | DCM | mild ID | beneficial effect | beneficial effect |
| 5, 4, F | Pat.1, CB, VII:11, [7] | 4 y | 3 | 15480 |  | no | no | No | no | beneficial effect | NA |
| 6, 4, F | Pat.2, #49591, MJ, VII:6, [7] | 4 y | 3 | 12240 |  | no | no | No | mild ID | beneficial effect | beneficial effect |
| 7, 4, M | Pat.3, JJ, VII:8, [7] | 4 y | 3 | 14760 |  | no | no | No | no | beneficial effect | NA |
| 8, 5, F | Pat.4, CV, [7] | early childhood | 3 | 10800 |  | DS | no | HCM | mild DD | beneficial effect | NA |
| 9, 6, F | Pat.1, #69842, [6] | 8 m | 2 | 6480 |  | PS | NA | HCM | no | beneficial effect | beneficial effect |
| 10, 7, F | Pat.2 (twin sister), [6] | 4 m | 2 | 180* |  | PS | NA | HCM | NA | NA | NA |
| 11, 7, F | Pat.3 (twin sister), [6] | 6 m | 2 | 240* |  | PS | NA | HCM | NA | NA | NA |
| 12, 8, M | Pat.1, #59029, 72545, [12] | 6 m | 2 | 5370* | Oligohydramnios | DS | no | HCM, DCM | mild DD | beneficial effect | beneficial effect |
| 13, 8, F | Pat.2, #59033, [12] | 4.5 m | 2 | 2160 | Oligohydramnios | DS | no | HCM | mild DD | beneficial effect | NA |
| 14, 8, F | Pat.3, #59036, [12] | 4 m | 2 | 3240 | Oligohydramnios | DS | no | HCM, DCM | mild DD | beneficial effect | NA |
| 15, 9, M | [33] | neonatal | 1 | 28* |  | PS | NA | NA | NA | NA | NA |
| 16, 10, M | [13] | 1 y | 2 | 6840 |  | no | NA | No | mild ID, mild DD | beneficial effect | NA |
| 17, 11,F | [34] | neonatal | 1 | 180* |  | DS | no | HCM | NA | no effect | NA |
| 18, 12,M | [15] | prenatal | 0 | 1* |  | PS | PS | HCM | NA | untreated | Beneficial effect |
| 19, 13, F | P2, [18] | neonatal | 1 | 70* | IGR, fetal rhythm abnormalities | PS | PS | HCM | no | untreated | NA |
| 20, 14, F | P3, [18] | 1 y | 2 | 2160* |  | PS | PS | HCM, DCM | NA | untreated | beneficial effect |
| 21, 15, F | P4, [18] | 18 m | 3 | 5400 |  | PS | no | HCM | no | untreated | no effect |
| 22, 16, F | P5, [18] | 9 y | 3 | 12600 |  | PS | no | HCM | no | NA | no effect |
| 23, 17,M | P6, [18] | 4 m | 2 | 7920 |  | PS | no | HCM | no | untreated | no effect |
| 24, 18, F | P7, [18] | 15 m | 3 | 630* |  | PS | NA | HCM | no | untreated | NA |
| 25, 19, F | P8, [18] | 1 y | 2 | 540* |  | DS | no | HCM | no | untreated | NA |
| 26, 20, F | Pt090, [19] | 2 w | 1 | 14 | IGR | PS | NA | HCM | NA | NA | NA |
| 27, 21, F | Pt025, [19] | 14 y | 4 | 5040 |  | PS | NA | HCM | NA | NA | NA |
| 28, 22,M | [21] | neonatal | 1 | 1* | IGR, fetal rhythm abnormalities | PS | PS | HCM, DCM | NA | NA | NA |
| 29, 23, F | P1, [22] | 2 d | 1 | 3180 |  | PS | DS | HCM | mild DD | no effect | NA |
| 30, 24,M | P2, [22] | 1 m | 1 | 90* | 32 Hbd: preterm uterus contractions | PS | DS | HCM, DCM | mild DD | untreated | NA |
| 31, 25,M | P3, [22] | 2 y | 3 | 3240 |  | no | no | no | no | NA | NA |
| 32, 26, F | I-1, [8] | neonatal | 1 | 150* | prematurely 26.7w due to pre-eclampsia | NA | NA | NA | NA | NA | NA |
| 33, 26, F | I-2, [8] | 2 m | 2 | 315* |  | DS | DS | HCM | severe DD | no effect | NA |
| 34, 26,M | I-3, [8] | 15 d | 1 | 270* |  | PS | NA | HCM | mild ID, severe DD | untreated | NA |
| 35, 26, F | I,4, [8] | 15 m | 3 | 3240 |  | NA | NA | NA | mild ID, mild DD | beneficial effect | NA |
| 36, 27,M | II-1, [8] | 12 y | 4 | 10440 |  | PS | NA | HCM | mild DD | NA | NA |
| 37, 27, F | II-2, [8] | 8 y | 3 | 9360 |  | PS | NA | HCM | mild ID, mild DD | untreated | NA |
| 38, 28, F | III-3, [8] | 2 d | 1 | 9* | born after preclampsia of the mother, 29w | NA | NA | NA | NA | untreated | NA |
| 39, 28, F | III-6, [8] | neonatal | 1 | 2* |  | PS | NA | HCM | NA | untreated | NA |
| 40, 28, F | III-7, [8] | neonatal | 1 | 180* |  | PS | NA | HCM, DCM | mild ID | no effect | NA |
| 41, 29, F | [35] | 6 y | 3 | 12240 |  | no | NA | No | no | NA | NA |
| 42, 30,M | This paper | prenatal | 0 | 2* | lissencephalopathy agenesis of the corpus callosum, IGR | PS | NA | DCM | NA | untreated | no effect |
| 43, 31,M | This paper | 3 m | 2 | 120* |  | PS | no | HCM | NA | untreated | NA |
| 44, 32, F | This paper | 7 y | 3 | 5040 |  | no | NA | No | no | no effect | no effect |
| 45, 33, F | This paper | 4 m | 2 | 540* |  | PS | NA | DCM | mild ID, severe DD | untreated | NA |
| 46, 33,M | This paper | 9 m | 2 | 3960 |  | DS | no |  | no | beneficial effect | NA |
| 47, 33,M | This paper | 7m | 2 | 720 |  | DS | no | HCM | mild ID, mild DD | beneficial effect | NA |
| 48, 34, F | This paper | neonatal | 1 | 2880 |  | DS | no | HCM, DCM | mild ID, mild DD | no effect | Beneficial effect |
| 49, 35, F | This paper | 1 y | 2 | 3960* |  | PS | no | HCM | mild ID, mild DD | NA | NA |
| 50, 36,M | This paper | neonatal | 1 | 1080 | hydronephrosis left, IGR | no | no | no | mild DD | NA | NA |
| 51, 37, F | This paper | 2 m | 2 | 180* |  | PS | NA | HCM, DCM | NA | untreated | NA |
| 52, 37,M | This paper | 10 y | 3 | 4680 |  | PS | no | HCM | mild ID, mild DD | beneficial effect | NA |
| 53, 38,M | This paper | 9 m | 2 | 270* |  | PS | no | HCM | no | untreated | NA |
| 54, 38,M | This paper | 9 m | 2 | 3210 |  | PS | PS | HCM | severe ID, mild DD | beneficial effect | NA |
| 55, 38,M | This paper | prenatal | 0 | 280 |  | PS | PS | HCM | mild ID, mild DD | beneficial effect | NA |
| 56, 39, F | This paper | neonatal | 1 | 1* | oligoidramnios, IGR, decreased child movements | PS | NA | HCM | NA | untreated | NA |
| 57, 40,M | This paper | neonatal | 1 | 28* |  | no | no | no | NA | no effect | NA |
| 58, 40,M | This paper | 6 m | 2 | 210* |  | NA | NA | NA | no | untreated | NA |
| 59, 41,M | This paper | 2 y | 3 | 13680 |  | PS | no | HCM | mild DD | no effect | NA |
| 60, 41,M | This paper | 9 y | 3 | 15840 |  | DS | NA | NA | no | no effect | NA |
| 61, 42, F | This paper | 2 m | 2 | NA |  | PS | NA | HCM | no | NA | NA |
| 62, 43, F | This paper | 2 y | 3 | 1080 |  | PS | NA | HCM | no | untreated | NA |
| 63, 44,M | This paper | neonatal | 1 | 3240 |  | PS | no | HCM | mild DD | beneficial effect | NA |
| 64, 45, F | This paper | 1 y | 2 | 6000 |  | PS | no | HCM | mild DD | beneficial effect | NA |
| 65, 46,M | This paper | infancy | 2 | 15840* |  | PS | NA | HCM, DCM | no | NA | NA |
| 66, 46,M | This paper | neonatal | 1 | 5760 |  | PS | no | HCM | no | no effect | NA |
| 67, 47,M | This paper | neonatal | 1 | 60* |  | PS | NA | HCM | severe DD | no effect | NA |
| 68, 48,M | This paper | 2 y | 3 | 1260 |  | PS | no | HCM, DCM | mild DD | beneficial effect | NA |
| 69, 49, F | This paper | 10 m | 2 | 1350 |  | PS | no | HCM, DCM | mild DD | beneficial effect | NA |
| 70, 50, F | This paper | 4 m | 2 | 2340 |  | no | no | no | mild ID, mild DD | beneficial effect | NA |

*0 = prenatal, 1 = neonatal (birth -28 days), 2 =>1-12 months, 3 = >12m -10y, 4 = adolescence, 5 = adult, 6 = na. DCM= Dilated Cardiomyopathy, DD=developmental delay, DS=developing symptom, HCM=Hypertrophic cardiomyopathy, ID=intellectual disability; PS= presenting symptom; NA= not available.
