# Supplementary figures and images for: Clinical, biochemical and genetic spectrum of 70 patients with ACAD9 deficiency: is riboflavin supplementation effective?
Source: Orphanet J Rare Dis. 2018 Jul 19;13:120. doi: 10.1186/s13023-018-0784-8 (PMC6053715; doi:10.1186/s13023-018-0784-8)

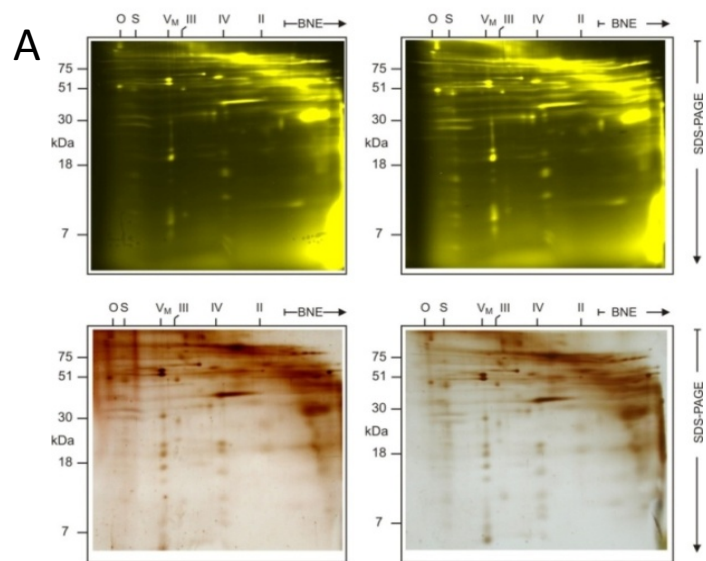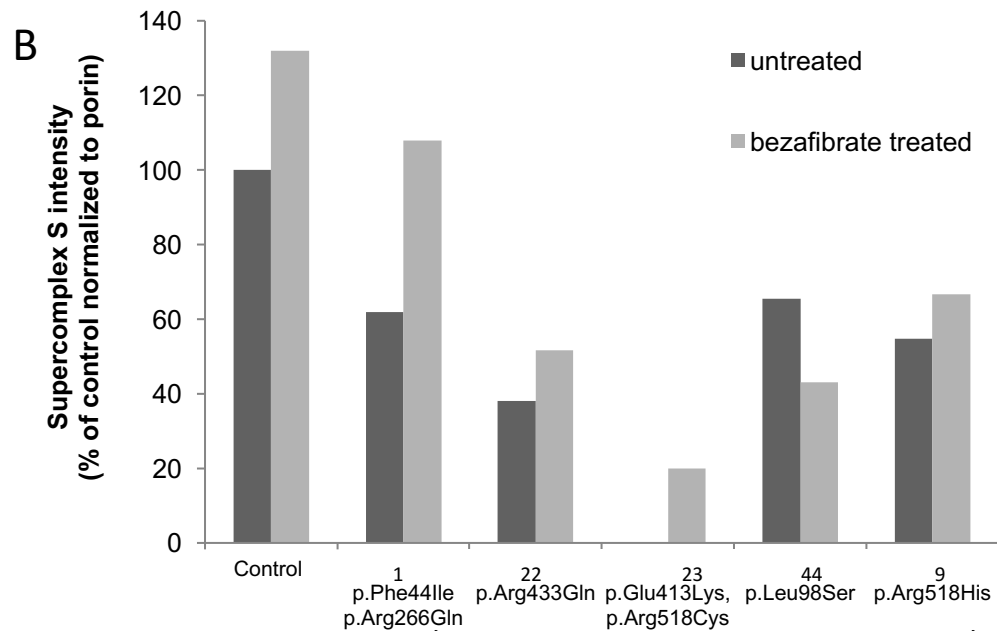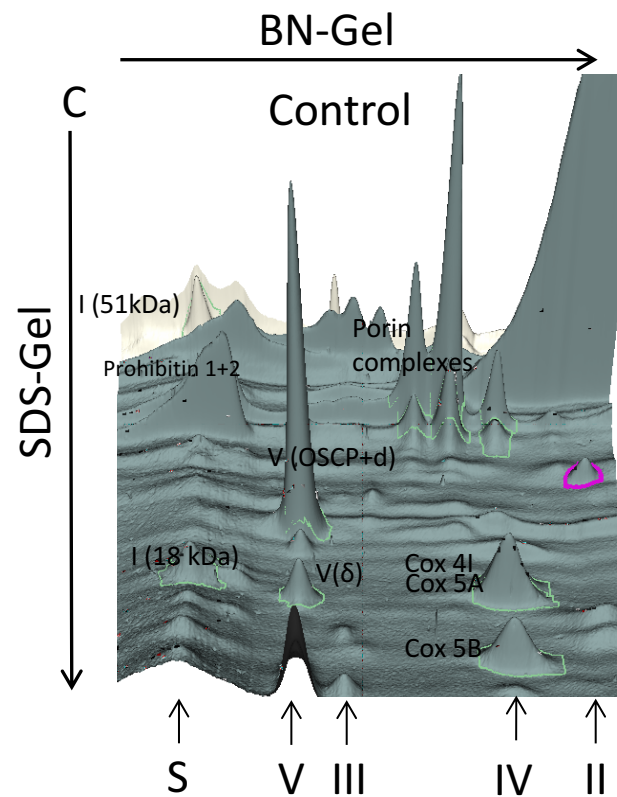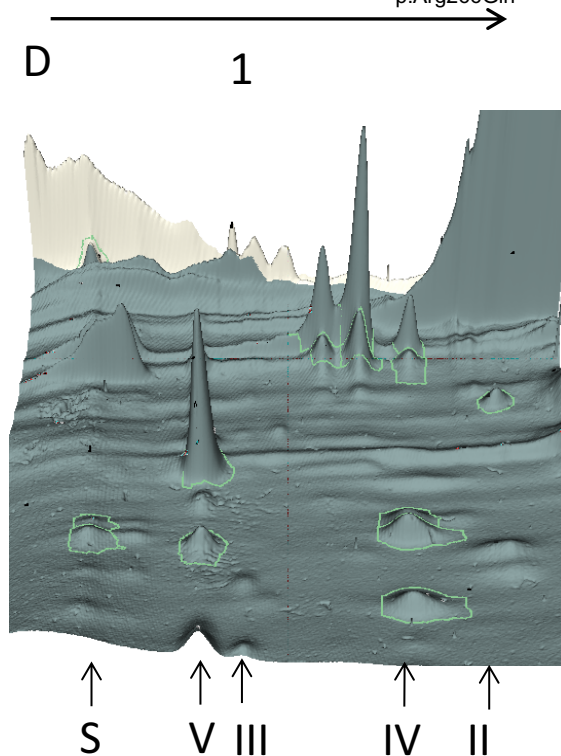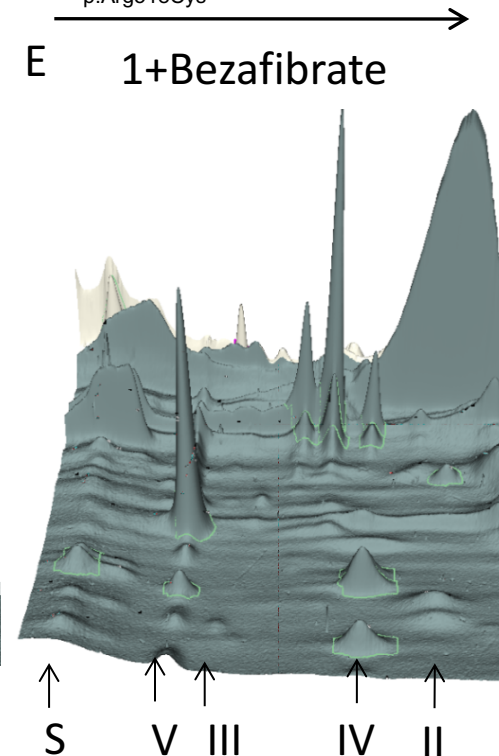

Supplement: Supplementary file 5 — Figure S1. Representative picture of Complex I assembly in fibroblasts of individual 1 (A, upper panels) Two-dimensional BN/SDS-PAGE separation and quantification of fluorescent-labelled mitochondrial complexes from 10 mg patient (left) and control fibroblasts (right) are shown. (A, lower panels) show silver stained 2 D gels. (B) Quantified Supercomplexes in 2D gels from control and patient fibroblast with and without bezafibrate treatment for 72 h. (C) Panoramaplots of 2D gels with assignment of signals used for quantification of complexes. Assignment of complexes: O, OGDC, oxoglutarate dehydrogenase complex; V, complex V or ATP synthase; III, complex III or cytochrome c reductase; IV, complex IV or cytochrome c oxidase; S, supercomplexes composed of respiratory chain complexes I, III, and IV. 2-D gels were scanned side by side for direct comparison and are shown as pseudocolors. (pdf). (PDF 1109 kb) [file 13023_2018_784_MOESM5_ESM.pdf]
